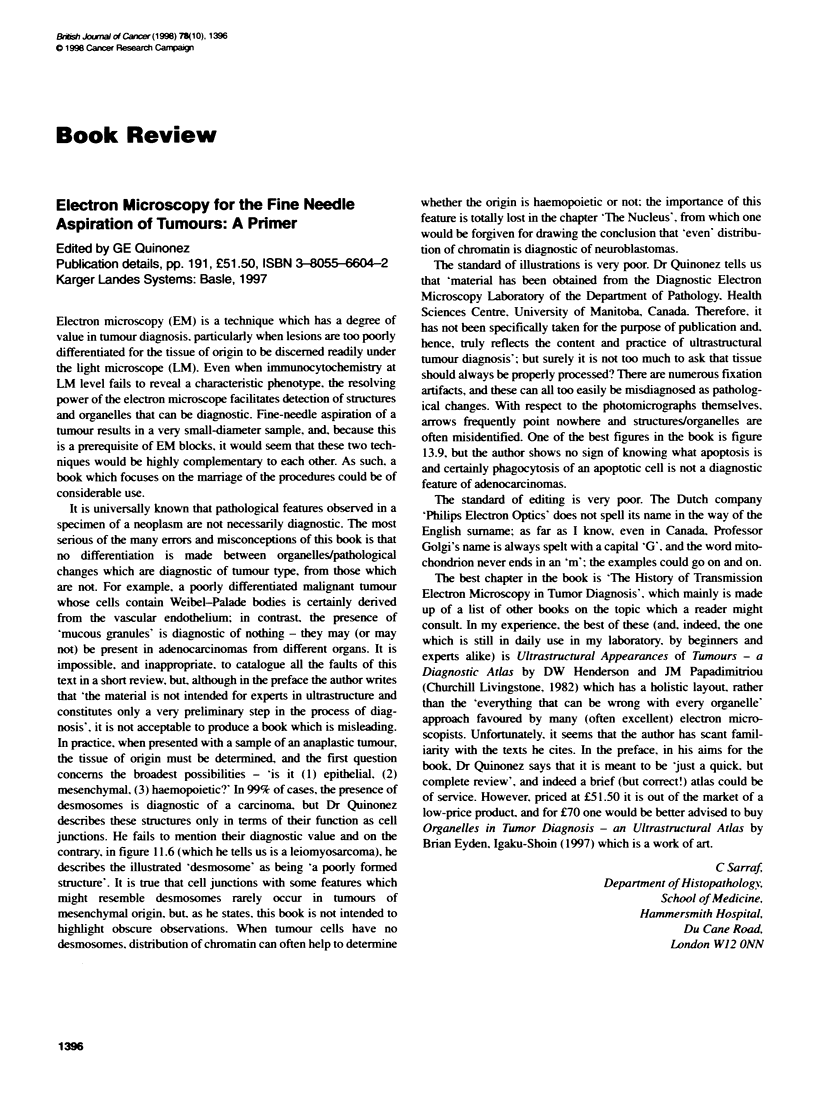# Electron Microscopy for the Fine Needle Aspiration of Tumours: A Primer

**Published:** 1998-11

**Authors:** C Sarraf


British Journal of Cancer (1998) 78(10), 1396

© 1998 Cancer Research Campaign

### Book Review

### Electron Microscopy for the Fine Needle Aspiration of Tumours: A Primer

Edited by GE Quinonez
Publication details, pp. 191, £51.50, ISBN 3–8055–6604–2
Karger Landes Systems: Basle, 1997

Electron microscopy (EM) is a technique which has a degree of value in tumour diagnosis, particularly when lesions are too poorly differentiated for the tissue of origin to be discerned readily under the light microscope (LM). Even when immunocytochemistry at LM level fails to reveal a characteristic phenotype, the resolving power of the electron microscope facilitates detection of structures and organelles that can be diagnostic. Fine-needle aspiration of a tumour results in a very small-diameter sample, and, because this is a prerequisite of EM blocks, it would seem that these two techniques would be highly complementary to each other. As such, a book which focuses on the marriage of the procedures could be of considerable use.

It is universally known that pathological features observed in a specimen of a neoplasm are not necessarily diagnostic. The most serious of the many errors and misconceptions of this book is that no differentiation is made between organelles/pathological changes which are diagnostic of tumour type, from those which are not. For example, a poorly differentiated malignant tumour whose cells contain Weibel–Palade bodies is certainly derived from the vascular endothelium; in contrast, the presence of 'mucous granules' is diagnostic of nothing – they may (or may not) be present in adenocarcinomas from different organs. It is impossible, and inappropriate, to catalogue all the faults of this text in a short review, but, although in the preface the author writes that 'the material is not intended for experts in ultrastructure and constitutes only a very preliminary step in the process of diagnosis', it is not acceptable to produce a book which is misleading. In practice, when presented with a sample of an anaplastic tumour, the tissue of origin must be determined, and the first question concerns the broadest possibilities – 'is it (1) epithelial, (2) mesenchymal, (3) haemopoietic?' In 99% of cases, the presence of desmosomes is diagnostic of a carcinoma, but Dr Quinonez describes these structures only in terms of their function as cell junctions. He fails to mention their diagnostic value and on the contrary, in figure 11.6 (which he tells us is a leiomyosarcoma), he describes the illustrated 'desmosome' as being 'a poorly formed structure'. It is true that cell junctions with some features which might resemble desmosomes rarely occur in tumours of mesenchymal origin, but, as he states, this book is not intended to highlight obscure observations. When tumour cells have no desmosomes, distribution of chromatin can often help to determine whether the origin is haemopoietic or not; the importance of this feature is totally lost in the chapter 'The Nucleus', from which one would be forgiven for drawing the conclusion that 'even' distribution of chromatin is diagnostic of neuroblastomas.

The standard of illustrations is very poor. Dr Quinonez tells us that 'material has been obtained from the Diagnostic Electron Microscopy Laboratory of the Department of Pathology, Health Sciences Centre, University of Manitoba, Canada. Therefore, it has not been specifically taken for the purpose of publication and, hence, truly reflects the content and practice of ultrastructural tumour diagnosis'; but surely it is not too much to ask that tissue should always be properly processed? There are numerous fixation artifacts, and these can all too easily be misdiagnosed as pathological changes. With respect to the photomicrographs themselves, arrows frequently point nowhere and structures/organelles are often misidentified. One of the best figures in the book is figure 13.9, but the author shows no sign of knowing what apoptosis is and certainly phagocytosis of an apoptotic cell is not a diagnostic feature of adenocarcinomas.

The standard of editing is very poor. The Dutch company 'Philips Electron Optics' does not spell its name in the way of the English surname; as far as I know, even in Canada, Professor Golgi's name is always spelt with a capital 'G', and the word mitochondrion never ends in an 'm'; the examples could go on and on.

The best chapter in the book is 'The History of Transmission Electron Microscopy in Tumor Diagnosis', which mainly is made up of a list of other books on the topic which a reader might consult. In my experience, the best of these (and, indeed, the one which is still in daily use in my laboratory, by beginners and experts alike) is *Ultrastructural Appearances* of *Tumours – a Diagnostic Atlas* by DW Henderson and JM Papadimitriou (Churchill Livingstone, 1982) which has a holistic layout, rather than the 'everything that can be wrong with every organelle' approach favoured by many (often excellent) electron microscopists. Unfortunately, it seems that the author has scant familiarity with the texts he cites. In the preface, in his aims for the book, Dr Quinonez says that it is meant to be 'just a quick, but complete review', and indeed a brief (but correct!) atlas could be of service. However, priced at £51.50 it is out of the market of a low-price product, and for £70 one would be better advised to buy *Organelles in Tumor Diagnosis – an Ultrastructural Atlas* by Brian Eyden, Igaku-Shoin (1997) which is a work of art.

*C Sarraf,*
*Department of Histopathology,*
*School of Medicine,*
*Hammersmith Hospital,*
*Du Cane Road,*
*London W12 0NN*